# Supplementary material for: A qualitative study of young migrants’ encounters with suicide and self-harm content, connecting and seeking help on social media
Source: Glob Ment Health (Camb). 2026 Apr 27;13:e90. doi: 10.1017/gmh.2026.10206 (PMC13161718; doi:10.1017/gmh.2026.10206)
Supplement: Basu et al. supplementary material [file S2054425126102064sup001.docx]

**ASSOCIATED STUDY: A qualitative study of young migrants’ encounters with suicide and self-harm content, connecting and seeking help on social media**

# Appendices

## Interview schedule

**General social media usage and motivations for being online**

1. To start, could you tell me about your experiences using social media – what sorts of things do you primarily use it for, what do you like and dislike about social media generally?

*Why do you feel that way?*

*What kind of things do you engage with online?*

**Understanding exposure or engagement with self-harm and suicide**

1. Could you tell me about any experiences you’ve had with **seeing** content about self-harm or suicide on social media.

***What type of content did you see?***

***On which platform(s)?***

***Was it text, images, videos? Who posted the content?***

1. How did seeing content about self-harm and/or suicide make you feel? How was it helpful or unhelpful?

***What type of content did you see?***

***On which platform(s)?***

***Was it text, images, videos? Who posted the content?***

1. Could you tell me about any experiences you’ve had with **engaging** with content about self-harm or suicide on social media.

*What type of content did you engage with?* ***On which platform(s)?***

***Was it text, images, videos? Who posted the content?*** *What made you engage with the content?*

1. How did engaging with the content about self-harm and/or suicide make you feel? How was it helpful or unhelpful?

*Do you think that it ever influenced your behaviour? How?*

1. Could you tell me about any experiences you’ve had **posting** content about self-harm or suicide on social media.

***What type of content did you post?***

***On which platform(s)?***

***Was it text, images, videos? Who posted the content?***

1. How did posting content about self-harm and/or suicide make you feel? How was it helpful or unhelpful?

*Did you feel better, worse or indifferent after posting the content? Why do you feel that way?*

**Using social media to maintain and form ties with community**

1. Do you feel that social media helps you to feel a sense of connection to your **country of origin**? This could be maintaining connections with family and friends, cultural practices, community events, etc.

*If yes:*

*What is an example of something you did on social media? Who did you reach out to? Which platform did you use? Why did you choose that platform? What was the outcome? Would you do it again? Do you think social media provides a good opportunity to form a connection?*

*If no:*

*Did you consider it at all? What made you not use social media?
Do you think social media provides a good opportunity to maintain connections?*

*Do you think social media has a negative effect on maintaining connections and why?*

1. Do you feel that social media helps you to feel a sense of connection to **Australia**? This could mean forming new relationships in Australia, learning more about the country, being aware of community events, etc.

*If yes:*

*What is an example of something you did on social media? Who did you reach out to? Which platform did you use? Why did you choose that platform? What was the outcome? Would you do it again?*

*Do you think social media provides a good opportunity to form new connections?*

*If no:*

*Did you consider it at all? What made you not use social media?
Do you think social media provides a good opportunity to form new connections?*

*Do you think social media has a negative effect on forming new connections and why?*

**Using social media to seek help for suicidal behaviour**

1. Have you used social media to **seek help** for suicidal thoughts, self-harm or attempted suicide?

*If yes:*

*What is an example of when you sought help using social media? Which platform did you use? Why did you choose that platform? Who did you seek help from (e.g. within Australia or from overseas)? What was the outcome? Would you do it again?*

*If no:*

*Did you consider social media as an option at all? What made you not use social media?
What have you used instead to seek help?*

## COREQ checklist

| **Domain 1: research team and reflexivity** | | |
| --- | --- | --- |
| **Personal characteristics** | | |
| 1. Interviewer/facilitator | Which author(s) conducted the interview or focus group? | Details provided in Researcher preparation and reflexivity section. |
| 2. Credentials | What were the researcher’s credentials? (e.g. PhD, MD) | Details provided on Title page. |
| 3. Occupation | What was their occupation at the time of the study? | Details provided on Title page. |
| 4. Gender | Was the researcher male or female? | The lead researcher and interviewer (AB) identifies as male. |
| 5. Experience and training | What experience or training did the researcher have? | Details provided in ‘Researcher preparation and reflexivity’ section. |
| **Relationship with participants** | | |
| 6. Relationship established | Was a relationship established prior to study commencement? | No |
| 7. Participant knowledge of the interviewer | What did the participants know about the researcher? (e.g. personal goals, reasons for doing the research) | Information provided in the discussed in the ‘Sample and recruitment’ section. |
| 8. Interviewer characteristics | What characteristics were reported about the interviewer/facilitator? (e.g. bias, assumptions, reasons and interests in the research topic) | Information provided in the discussed in the ‘Sample and recruitment’ section. |
| **Domain 2: Study design** | | |
| **Theoretical framework** | | |
| 9. Methodological orientation and theory | What methodological orientation was stated to underpin the study? (e.g. grounded theory, discourse analysis, ethnography, phenomenology, content analysis) | Reflexive thematic analysis approach explained in the ‘Data collection and analysis’ section. |
| **Participant selection** | | |
| 10. Sampling | How were participants selected? (e.g. purposive, convenience, consecutive, snowball) | Sampling approach is discussed in the ‘Sample and recruitment’ section. |
| 11. Method of approach | How were participants approached? (e.g. face to face, telephone, mail, e-mail) | Recruitment approach is discussed in the ‘Sample and recruitment’ section. |
| 12. Sample size | How many participants were in the study? | Sample size is discussed in the ‘Results’ section. |
| 13. Non-participation | How many people refused to participate or dropped out? Reasons? | N/A |
| **Participant selection** | | |
| 14. Setting of data collection | Where was the data collected? (e.g. home, clinic, workplace) | Reported in the ‘Data collection and analysis’ section. |
| 15. Presence of non-participants | Was anyone else present besides the participants and researchers? | No |
| 16. Description of sample | What are the important characteristics of the sample? (e.g. demographic data, date) | Relevant participant characteristics are presented in the ‘Results’ section. |
| **Data collection** | | |
| 17. Interview guide | Were questions, prompts, guides provided by the authors? Was it pilot tested? | Details of the interview guide are available in Appendix A. |
| 18. Repeat interviews | Were repeat interviews carried out? If yes, how many? | N/A |
| 19. Audio/visual recording | Did the research use audio or visual recording to collect the data? | Details of audio-recording and transcription is described in the ‘Data collection and analysis’ section. |
| 20. Field notes | Were field notes made during and/or after the interview or focus group? | This is included in the ‘Data collection and analysis’ section. |
| 21. Duration | What was the duration of the interviews or focus group? | This is included in the ‘Data collection and analysis’ section. |
| 22. Data saturation | Was data saturation discussed? | Data saturation wasn't considered an appropriate measure of data quality for the present study. |
| 23. Transcripts returned | Were transcripts returned to participants for comment and/or correction? | No |
| **Domain 3: Analysis and findings** | | |
| **Data analysis** | | |
| 24. Number of data coders | How many data coders coded the data? | Details of the coding approach is discussed in the ‘Data collection and analysis’ section. |
| 25. Description of the coding tree | Did authors provide a description of the coding tree? | Details of the coding approach is discussed in the ‘Data collection and analysis’ section. |
| 26. Derivation of themes | Were themes identified in advance or derived from the data? | Themes were derived from the data and outlined in the ‘Results’ section. |
| 27. Software | What software, if applicable, was used to manage the data? | Details of the software used is documented in the ‘Data collection and analysis’ section. |
| 28. Participant checking | Did participants provide feedback on the findings? | No |
| **Reporting** | | |
| 29. Quotations presented | Were participant quotations presented to illustrate the themes/findings? Was each quotation identified? (e.g. participant number) | Yes, illustrative quotes are provided throughout the results section and are attributed based on the participant’s ID. |
| 30. Data and findings consistent | Was there consistency between the data presented and the findings? | See ‘Results’ section. |
| 31. Clarity of major themes | Were major themes clearly presented in the findings? | See ‘Results’ section. |
| 32. Clarity of minor themes | Is there a description of diverse cases or discussion of minor themes? | See ‘Results’ section. |
